# Supplementary material for: Role of Defects of Carbon Nanomaterials in the Detection of Ovarian Cancer Cells in Label-Free Electrochemical Immunosensors
Source: Sensors (Basel). 2023 Jan 18;23(3):1131. doi: 10.3390/s23031131 (PMC9919683; doi:10.3390/s23031131)
Supplement: Supplementary file 1 [file sensors-23-01131-s001.zip › sensors-2131185-supplementary.pdf]

SUPPORTING INFORMATION

Role of Defects of Carbon Nanomaterials in the Detection of Ovarian Cancer Cells in Label-Free Electrochemical Immunosensors

Nattharika Runprapan [1], Fu-Ming Wang [1,2,3,4,*], Alagar Ramar [1] and Chiou-Chung Yuan [5]

1 Graduate Institute of Applied Science and Technology, National Taiwan University of Science and Technology, Taipei 106, Taiwan
2 R&D Center for Membrane Technology, Chung Yuan Christian University, Taoyuan 320, Taiwan
3 Sustainable Energy Center, National Taiwan University of Science and Technology, Taipei 106, Taiwan
4 Department of Chemical Engineering, Chung Yuan Christian University, Taoyuan 320, Taiwan
5 Department of Obstetrics and Gynecology, Cheng Hsin General Hospital, Taipei City 112, Taiwan
* Correspondence: mccabe@mail.ntust.edu.tw; Tel.: +886-2-27303755; Fax: +886-2-27303733

Table of content

Entry	Table of content	Page No
**Figure S1**	CV of electrodeposition of (a) 50AuNPs@MWCNTs, (b) 50AuNPs@VGCFs, (c) 50AuNPs@KS4, and (d) 50AuNPs@SP.	S2
**Figure S2**	CV response of different cycles 10, 30, and 50 cycles of (a) AuNPs@MWCNTs, (b) AuNPs-VGCFs, (c) AuNPs@KS4, and (d) AuNPs@SP. All experiments were examined by 0.1 M KCl/5 mM $[Fe(CN)]^{3-/4-}$ (0.1 X PBS).	S3
**Table S1**	I_D/I_G ratio of carbon nanomaterials.	S3
**Figure S3**	XRD patterns of pristine and oxidized (a) MWCNTs, (b) VGCFs, (c) KS4, and (d) SP.	S4
**Table S2**	Crystallites size unmodified and acid treatment of carbon nanomaterials by Scherrer equation.	S4
**Figure S4**	XPS spectra of Au element in composite (a) 50AuNPs@MWCNTs, (b) 50AuNPs@VGCFs, (c) 50AuNPs@KS4, and (d) 50AuNPs@SP.	S5
**Figure S5**	CV profile (a) VGCFs, (c) KS4, (e) SP, and EIS spectra (inset: model equivalent circuit) (b) VGCFs, (d) KS4, and (f) SP of layer-by-layer immunosensors in different carbon nanomaterials in in 5 mM $[Fe(CN)_6]^{3-/4-}$ /0.1 M KCl (pH 5.5, 0.1 X PBS) with CV: potential range (E) is 0.4 to 0.8 V, scan rate is 10 mVs^{-1}. EIS: scan from f_i = 200 kHz to f_f = 10.00 mHz, sinus amplitude $V_a \approx$ 10 mV.	S6
**Figure S6**	Resistance of fabrication step networks of layer-by-layer construction of immunosensor in in 5 mM $[Fe(CN)_6]^{3-/4-}$/0.1 M KCl (pH 5.5, 0.1 X PBS) with EIS: scan from f_i = 200 kHz to f_f = 10.00 mHz, sinus amplitude $V_a \approx$ 10 mV.	S7

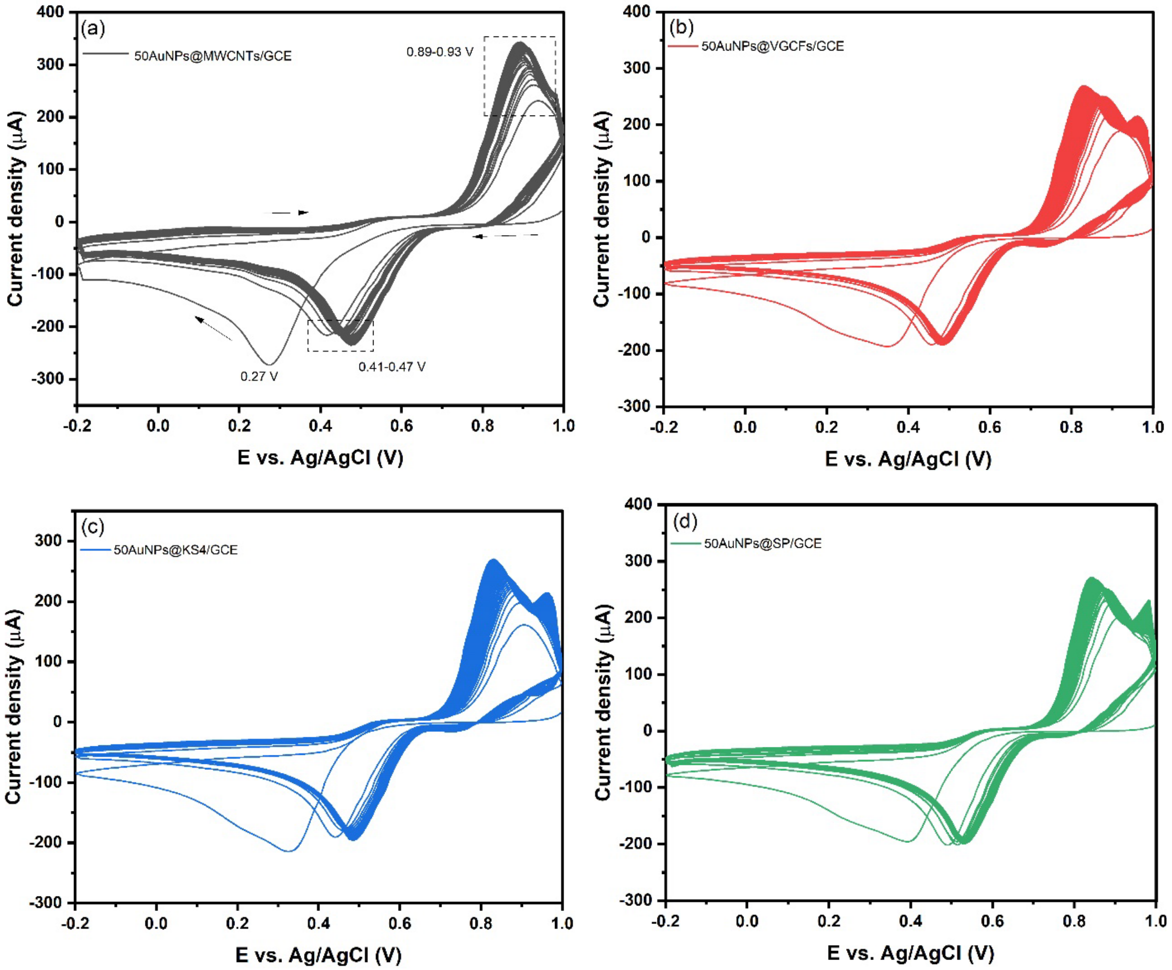

Figure S1. CV of electrodeposition of (a) 50AuNPs@MWCNTs, (b) 50AuNPs@VGCFs, (c) 50AuNPs@KS4, and (d) 50AuNPs@SP.

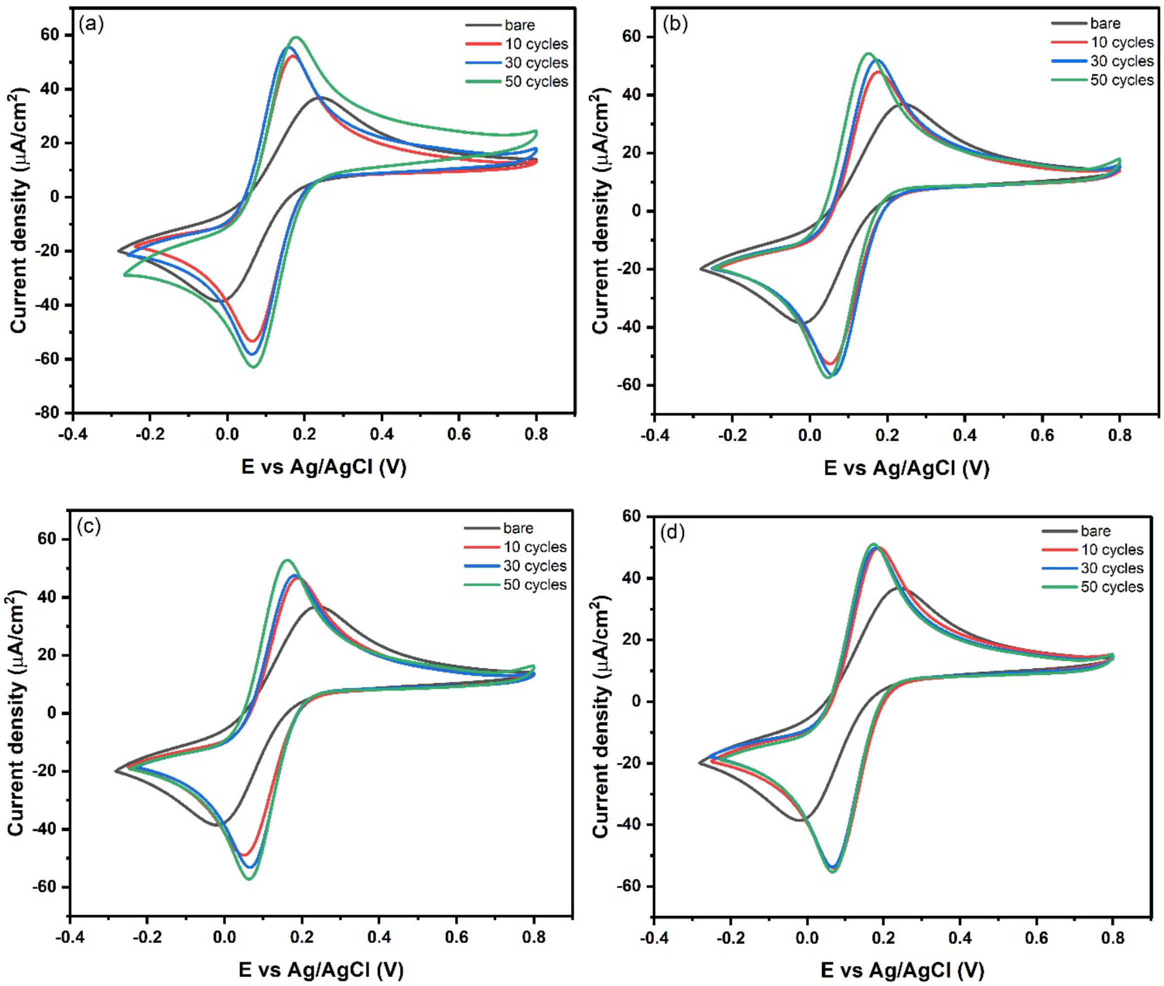

Figure S2. CV response of different cycles 10, 30, and 50 cycles of (a) AuNPs@MWCNTs, (b) AuNPs-VGCFs, (c) AuNPs@KS4, and (d) AuNPs@SP. All experiments were examined by 0.1 M KCl/5 mM $[Fe(CN)]^{3-/4-}$ (0.1 X PBS).

Table S1. I_D/I_G ratio of carbon nanomaterials.

samples	pristine	oxidized	I_D/I_G
	Position of peak D and G band		
MWCNT	1341, 1574	1339, 1573	1.190, 1.249
VGCFs	1345, 1575	1336, 1516	0.203, 0.272
KS4	1335, 1565	1345, 1569	0.240, 0.472
SP	1349, 1587	1345, 1581	1.122, 1.217

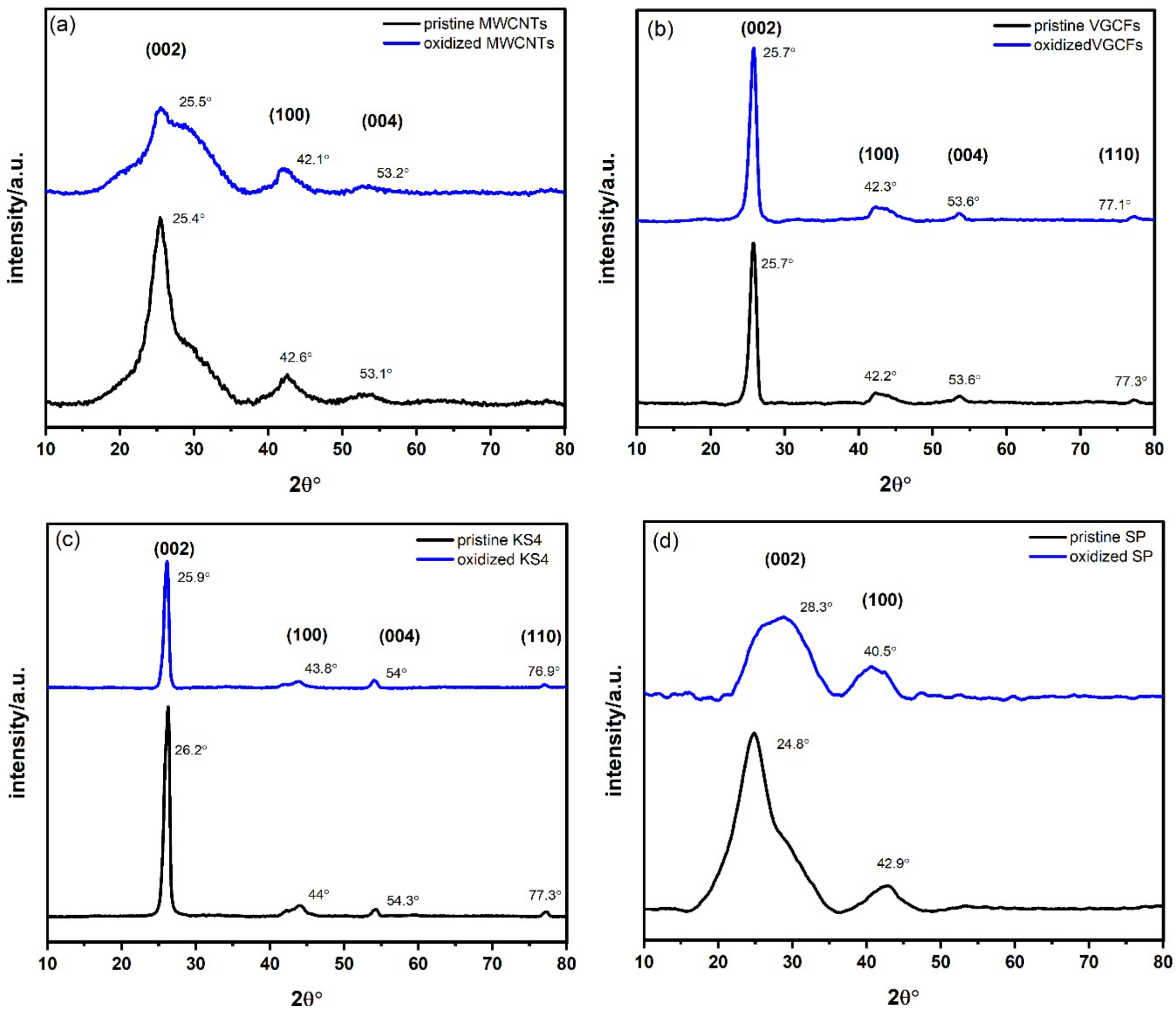

Figure S3. XRD patterns of pristine and oxidized (a) MWCNTs, (b) VGCFs, (c) KS4, and (d) SP.

Table S2. Crystallites size unmodified and acid treatment of carbon nanomaterials by Scherrer equation.

Materials	Crystallite size (nm)	
	untreated	treated
MWCNTs	1.73	1.67
VGCFs	6.46	6.30
KS4	9.58	9.12
SP	1.64	1.49

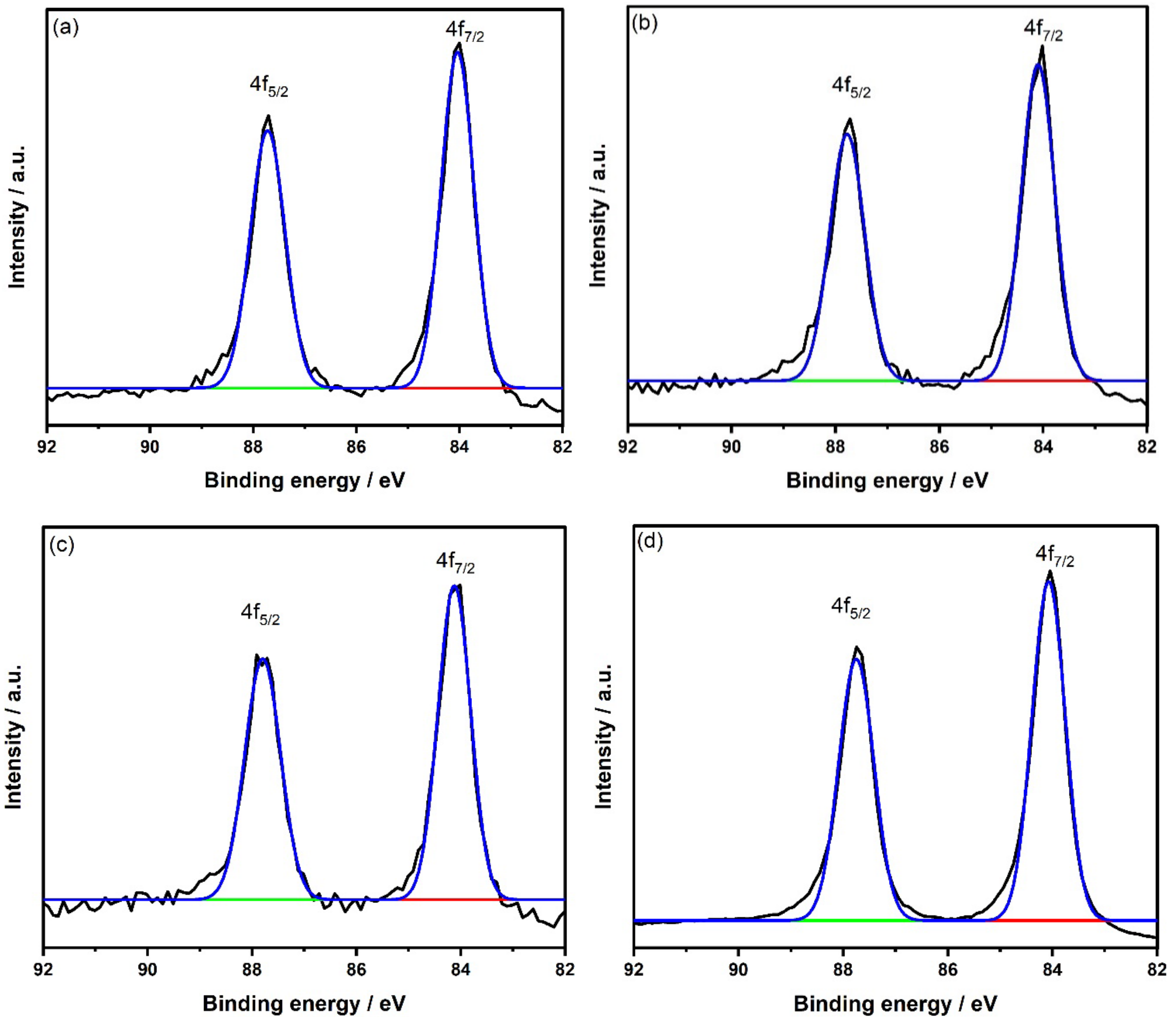

Figure S4. XPS spectra of Au element in composite (a) 50AuNPs@MWCNTs, (b) 50AuNPs@VGCFs, (c) 50AuNPs@KS4, and (d) 50AuNPs@SP.

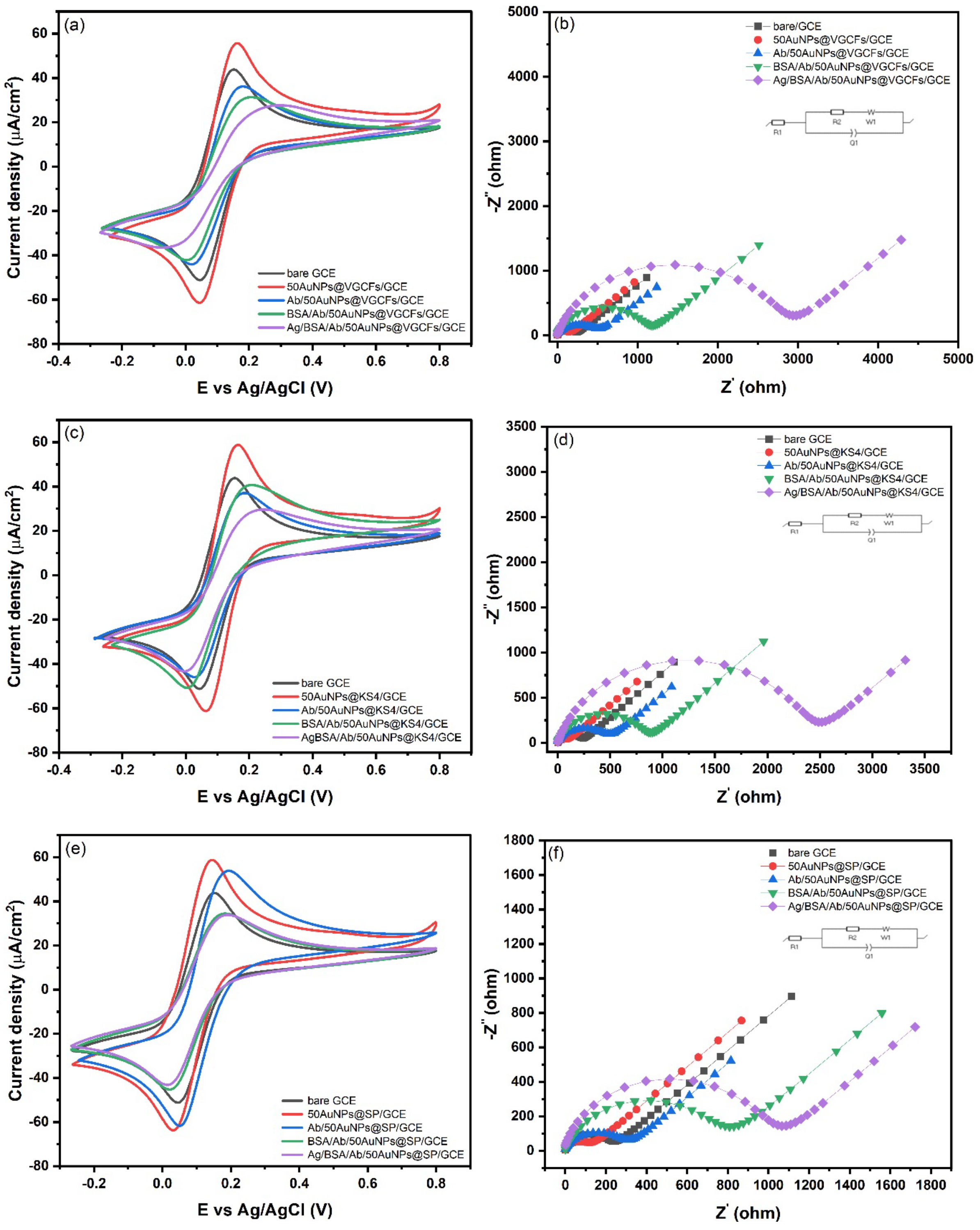

Figure S5. CV profile (a) VGCFs, (c) KS4, (e) SP, and EIS spectra (inset: model equivalent circuit) (b) VGCFs, (d) KS4, and (f) SP of layer-by-layer immunosensors in different carbon nanomaterials in in 5 mM $[Fe(CN)_6]^{3-/4-}$ /0.1 M KCl (pH 5.5, 0.1 X PBS) with CV: potential range (E) is 0.4 to 0.8 V, scan rate is 10 mVs^{-1}. EIS: scan from f_i = 200 kHz to f_f = 10.00 mHz, sinus amplitude $V_a \approx$ 10 mV.

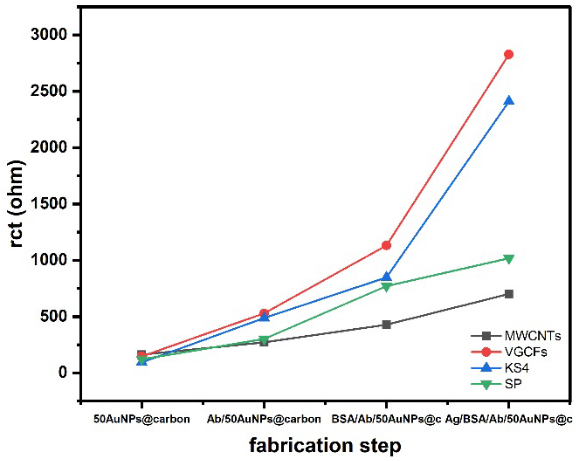

Figure S6. Resistance of fabrication step networks of layer-by-layer construction of immunosensor in in 5 mM $[Fe(CN)_6]^{3-/4-}$/0.1 M KCl (pH 5.5, 0.1 X PBS) with EIS: scan from f_i = 200 kHz to f_f = 10.00 mHz, sinus amplitude $V_a \approx$ 10 mV.
